# Pediatric non-diabetic ketoacidosis: a case-series report

**DOI:** 10.1186/s12887-017-0960-3

**Published:** 2017-12-19

**Authors:** Ke Bai, Yueqiang Fu, Chengjun Liu, Feng Xu, Min Zhu

**Affiliations:** 1 Intensive Care Unit, Children’s Hospital of Chongqing Medical University, Ministry of Education Key Laboratory of Children Development and Disorders, China International Science and Technology Cooperation base of Child development and Critical Disorders, Chongqing Key Labortory of Pediatrics, 136 Zhongshang Er Road, Yuzhong District, Chongqing, 400014 People’s Republic of China; 2 Department of Endocrinology, Children’s Hospital of Chongqing Medical University, Ministry of Education Key Laboratory of Children Development and Disorders, China International Science and Technology Cooperation base of Child development and Critical Disorders, Chongqing Key Labortory of Pediatrics, Chongqing, 400014 People’s Republic of China

**Keywords:** Non-diabetic ketoacidosis, Glucose, Insulin, Bicarbonate, Continuous renal replacement therapy

## Abstract

**Background:**

This study is to explore the clinical characteristics, laboratory diagnosis, and treatment outcomes in pediatric patients with non-diabetic ketoacidosis.

**Methods:**

Retrospective patient chart review was performed between March 2009 to March 2015. Cases were included if they met the selection criteria for non-diabetic ketoacidosis, which were: 1) Age ≤ 18 years; 2) urine ketone positive ++ or >8.0 mmol/L; 3) blood ketone >3.1 mmol/L; 4) acidosis (pH < 7.3) and/or HCO_3_ < 15 mmol/L; 5) random blood glucose level < 11.1 mmol/L. Patients who met the criteria 1, 4, 5, plus either 2 or 3, were defined as non-diabetic ketoacidosis and were included in the report.

**Results:**

Five patients with 7 episodes of non-diabetic ketoacidosis were identified. They all presented with dehydration, poor appetite, and Kussmaul breathing. Patients treated with insulin plus glucose supplementation had a quicker recovery from acidosis, in comparison to those treated with bicarbonate infusion and continuous renal replacement therapy. Two patients treated with bicarbonate infusion developed transient coma and seizures during the treatment.

**Conclusion:**

Despite normal or low blood glucose levels, patients with non-diabetic ketoacidosis should receive insulin administration with glucose supplementation to correct ketoacidosis.

## Background

Diabetic ketoacidosis is a common cause of metabolic acidosis. However, ketoacidosis is not always accompanied with high blood glucose and diabetes. Some patients with ketoacidosis may have no medical history of diabetes and have normal or low blood glucose levels. These patients with non-diabetic ketoacidosis may also experience severe complications or even death [1]. There has been very limited research involving patients with non-diabetic ketoacidosis, especially those in the pediatric age-group.

Diabetic ketoacidosis requires intravenous fluid replacement, insulin injection, correction of electrolyte imbalance, and possible bicarbonate administration in patients with severe acidosis, as well as prompt treatment for its etiology such as infection or insufficient insulin administration [2]. Patients with non-diabetic ketoacidosis also have increased ketone bodies, but they usually have normal or low blood glucose levels. Currently, there is no general consensus on the treatment for non-diabetic ketoacidosis. Several previous case reports have described non-diabetic ketoacidosis [3, 4], but they usually focused on the exploration of original disorders, while failing to describe the initial treatment for stabilization of these patients. We believe that the initial treatment for stabilization of ketoacidotic patients is a critical step that allows for further investigation of underlying cause of the disorder.

Herein, we present a case-series comprising of five children with non-diabetic ketoacidosis, with the purpose to explore the clinical characteristics, laboratory diagnosis, and treatment outcomes in pediatric patients with non-diabetic ketoacidosis.

## Methods

A retrospective case-series report has been presented. The study protocol was approved by the Children’s Hospital of Chongqing Medical University institutional review board and written informed consent was obtained from all the patients and their guardians. Hospital electronic medical records pertaining to the period between March 2009 and March 2015 were searched for cases qualifying the selection criteria.

### Case selection criteria

There are no previously reported diagnostic criteria for non-diabetic ketoacidosis. Our selection criteria were based on the consensus guideline for pediatric diabetic ketoacidosis [5], except that the blood glucose level was low or normal: 1) age ≤ 18 years; 2) urine ketones positive ++ or >8.0 mmol/L; 3) blood ketone level > 3.1 mmol/L; 4) acidosis (pH < 7.3 or HCO_3_ < 15 mmol/L); 5) random blood glucose level < 11.1 mmol/L. Patients who met the criteria 1, 4, 5, plus either 2 or 3 were defined as cases of pediatric non-diabetic ketoacidosis and included in the current report.

### Data collection

Data collected pertained to the clinical presentation, laboratory test results, brain magnetic resonance imaging (MRI), blood tandem mass spectrometry and urine gas chromatography mass spectrometry analyses, genetic test result, treatment approach (insulin, bicarbonate, continuous renal replacement therapy), and outcomes (time required for recovery from acidosis). Ketone was detected with quantitative method (Abbott, USA),

## Results

Five patients met the selection criteria for pediatric non-diabetic ketoacidosis. One of these 5 patients was hospitalized three times. Thus, a total of 7 episodes of non-diabetic ketoacidosis are captured in this report. All patients had signs of dehydration, poor appetite, and Kussmaul breathing. The baseline demographics, clinical presentation, etiology, and initial laboratory test results are summarized in Table 1.

**Table 1:** Characteristics of reported cases

	Gender	Weight (kilogram)	Degree of dehydration	Age (year)	Etiology	Neurological signs			Blood glucose (mmol/L)	Anion gap	Serum	Serum	Serum	Ketones	
						Drowsiness	Coma	Seizure			Cl-mmol/L	K + mmol/L	HCO_3_(mmol/L)	Blood (mmol/L)	Urine
Case 1	Female	≤ 10	mild	< 1	URI	N/A	+	+	2.0	25.3	112.3	3.5	3.4	N/A	+++
Case 2	Female	10–20	mild	1–5	Sepsis	+	–	–	2.1	18.5	115.7	2.6	4.5	N/A	+++
Case 3	Male	≤ 10	mild	1–5	Gastroenteritis	+	–	–	6.4	27.6	105.8	3.8	3	5.1	+++
Case 4	Male	10–20	mild	1–5	URI	+	–	–	4.5	37.3	96.9	4.5	< 3	>6	+++
Case 5(1)	Male	≤ 10	mild	< 1	Gastroenteritis	N/A	+	+	4.3	30.1	105.8	2.7	3.8	N/A	+++
(2)		≤ 10	mild	< 1	Gastroenteritis	+	–	–	5.1	34.4	98.2	4.6	< 3	>6	+++
(3)		10–20	mild	1–5	Gastroenteritis	+	–	–	4.7	27.5	103.9	5.0	4.8	4.9	+++

*URI* upper respiratory tract infection

N/A record missing

One patient (case 5) had increased blood 3-hydroxybutyrate acid carnitine as well as urine 3-hydroxybutyric acid, 3-hydroxy acid, and acetyl glycine levels. Genetic testing revealed a mutation in the T2 gene (456 C > T), and the patient was diagnosed as a case of β-ketothiolase deficiency. This child had sustained three episodes of ketoacidosis and had more severe clinical presentation including seizures during the first episode. This child also had symmetric abnormal signals in the bilateral lenticular nucleus in the brain MRI study, suggesting local edema.

Treatment included intravenous fluid resuscitation, insulin, glucose supplementation, bicarbonate infusion, and continuous renal replacement therapy (CRRT) (Table 2). All children survived till hospital discharge. There was no serious hypokalemia or hypoglycemia event.

**Table 2:** Treatment details and outcomes

	Dosage of 5% NaHCO_3_ infused (ml/kg)^a^	Dosage of insulin infused (unit/kg/h)^b^	Duration of insulin infusion (hour)	Dosage of glucose / insulin infused (gram/unit)	CRRT duration (hour)^c^	Time required for acidosis correction (hour)			
						From diagnosis	after stopping NaHCO_3_	After stopping insulin	After stoppage of CRRT
Case 1	85	–	–	–	–	186	3	–	–
Case 2	5	0.076	21	4.6	–	48	30	1	–
Case 3	20	0.083	20	5.7	–	116	44	< 1	–
Case 4	23	0.081	28	5.3	9	122	24	< 1	10
Case 5 (1)	24	0.079	8	4.5	17	89	79	< 1	36
(2)	2.5	0.078	42	5.6	–	60	54	< 1	–
(3)	3	0.082	17.5	5.2	–	21	18	< 1	–

^a^Biocarbonate was infused by diluting biocarbonate with 5% dextrose as a ratio of 1: 2.5 to a 1.4% isotonic solution

^b^Insulin was infused as a rate of 0.05–0.1 unit/kg/h

^c^ *CRRT* continuous renal replacement therapy

## Discussion

In this case-series report, we analyzed 7 episodes of pediatric non-diabetic ketoacidosis. The treatment approach to correct metabolic disorders in children with non-diabetic ketoacidosis is discussed.

### Diagnosis of pediatric non-diabetic ketoacidosis

The diagnosis of ketoacidosis is based on laboratory tests. Normal or low blood glucose level, low bicarbonates, high anion gap metabolic acidosis, and ketone bodies in blood or urine are consistent with the diagnosis of non-diabetic ketoacidosis. After stabilization of patients, further investigations are mandated for determining the underlying cause of non-diabetic ketoacidosis. The causes of non-diabetic ketoacidosis include severe starvation, organic acidemia (such as β-ketothiolase deficiency, propionic acidemia, methylmalonic acidemia, hyperglycinemia), glycogen storage disease, and gluconeogenesis disorders [6]. One child in our case series had a genetic mutation in T2 gene and was diagnosed as a case of β-ketothiolase deficiency. Patients with this disorder can have impaired ketone metabolism and ketone accumulation in the body.

### Treatment for pediatric non-diabetic ketoacidosis

Non-diabetic ketoacidosis is treated similarly as diabetic ketoacidosis, but it also requires some unique approaches. The treatment approach mainly consists of the following four components:

### Early glucose supplementation

The characteristics of diabetic ketoacidosis include high blood glucose, ketone accumulation, and acidosis. The high blood glucose is due to insulin deficiency or insulin resistance. Treatment should aim to limit glucose intake with concomitant insulin supplementation [2].

Patients with non-diabetic ketoacidosis commonly have normal or low blood glucose level (Table 1). Early glucose supplementation to maintain blood glucose level at a high-normal value could limit fatty acid oxidation and decrease production of ketone bodies [7]. This is an important aspect of the treatment of non-diabetic ketoacidosis that is different from that of diabetic ketoacidosis.

### Insulin administration

Insulin is required for glucose metabolism but it also inhibits lipolysis and stimulates fat synthesis and storage, thereby decreasing free fatty acid production. Free fatty acids are the main source for production of ketone bodies. Supplementation of insulin can inhibit lipolysis and ketone production, as well as decrease glucagon secretion, and is thus the main treatment for diabetic ketoacidosis [8].

In the 7 episodes of non-diabetic ketoacidosis reported here, insulin administration could successfully correct acidosis. One patient (case 1, Table 2) who was not treated with insulin infusion took the longest time in recovering from acidosis, despite the patient receiving the highest amount of bicarbonate infusion. The most severe case of acidosis (case 5, 2nd episode, Tables 1 and 2, serum bicarbonate <3 mmol/L) had received moderate amounts of bicarbonate infusion but prolonged insulin administration. He had relatively short recovery time from acidosis. This suggests the efficacy of insulin treatment in non-diabetic ketoacidosis. However, use of insulin can lead to hypoglycemia. In children with non-diabetic ketoacidosis, their blood glucose levels are usually low or normal (Table 1). Insulin should be administered together with glucose in a ratio of 4–6 g glucose for every 1 unit of insulin, with frequent blood glucose monitoring, in order to maintain a high-normal glucose value, since patients with non-diabetic ketoacidosis had high risk for hypoglycemia [9].

### Bicarbonate infusion

In a ketoacidotic state, bicarbonate ions can be lost after their binding with the accumulated ketone bodies, followed by their excretion through the kidney. This is the rationale for bicarbonate supplementation in these patients. However, administration of bicarbonate may not necessarily correct acidosis; it may rather aggravate acidosis and hypoxia in the central nervous system, exacerbate hypokalemia, change serum calcium concentration, and increase plasma osmolality [10, 11]. The infusion of biocarbonate increases the blood pCO_2_, which can cross the blood brain barrier faster than the biocarbonate. Increased pCO_2_ in the brain would result in paradoxical fall in cerebral pH. Biocarbonate infusion could increase blood pH, which induces intracellular shift of potassium and lead to hypokalemia. Alkalosis can also promote calcium binding to proteins and decrease free calcium level. The sodium infused together with biocarbonate could increase body sodium load and plasma osmolality. Thus, bicarbonate infusion in DKA is only suggested to patients with severe acidosis [5].

Similarly, infusion of biocarbonate in patients with non-diabetic ketoacidosis might not achieve satisfactory results. For example, out of the 7 episodes of non-diabetic ketoacidosis described in this report, two children who received the highest dose of sodium bicarbonate (case 1 and 1st episode of Case 5; Tables 1 and 2) had altered mental status and seizures during the treatment. The child with highest biocarbonate infusion (case 1, 85 mL/kg) required the longest time to correct acidosis. Both of them did not show any rapid reversal of acidosis. On the contrary, one patient (2nd episode in Case 5; Tables 1 and 2) with severe acidosis (serum bicarbonate <3 mmol/L) who received the lowest amount of bicarbonate infusion had a relatively shorter recovery time from acidosis. These findings suggest that, similar to diabetic ketoacidosis, the treatment of non-diabetic ketoacidosis should also be careful with the bicarbonate infusion. Bicarbonate should only be considered when there is severe acidosis and unstable hemodynamic signs after initial fluid resuscitation [6]. Bicarbonate can be administered in the form of 5% sodium bicarbonate solution in a dosage of 1~2 mL/kg administered over 1 hour [9].

### Continuous renal replacement therapy

Ketone bodies (acetoacetate, β- hydroxybutyric acid, and acetone) are small molecules and can be eliminated through CRRT. However, the use of CRRT in diabetic ketoacidosis has not been well studied, with only a few cases having been reported [12, 13]. Among the current cases, 2 cases of non-diabetic ketoacidosis who received CRRT showed only partial correction of acidosis. Their acidosis was corrected 10–36 h after stopping CRRT, which suggests that CRRT may not be efficacious in these patients. The poor efficacy could be explained by the fact that CRRT can only eliminate the existing ketone bodies, and does not have any effect on the production of ketone bodies. Once CRRT stops, the concentration of ketone bodies is bound to increase again with concomitant acidosis.

## Conclusion

In summary, our current analysis suggests that early identification of children with potential ketoacidosis (poor appetite, Kussmaul respiration, and rotten apple smell in the breath), prompt laboratory analysis, and early insulin administration with glucose supplementation can effectively correct ketoacidosis. Bicarbonate administration and CRRT have limited efficacy and should be used cautiously. Further studies are required to assess the optimal therapeutic approach to pediatric non-diabetic ketoacidosis.

## Abbreviations

CRRT: Continuous renal replacement therapy
MRI: Magnetic resonance imaging
